# A thymol-based blend of botanicals reduces *Salmonella* induced inflammation by altering key inflammatory signaling intermediates differentially depending on dose and in a manner distinct from in-feed antibiotics

**DOI:** 10.1016/j.psj.2025.105713

**Published:** 2025-08-21

**Authors:** Casey N. Johnson, Giulia Giovagnoni, Famatta Perry, Benedetta Tugnoli, Denise Caldwell, Christina Swaggerty, Haiqi He, Andrea Piva, Ester Grilli, J. Allen Byrd, Ryan J. Arsenault

**Affiliations:** a Food and Feed Safety Research Unit, Southern Plains Agricultural Research Center, Department of Agriculture – Agricultural Research Service, College Station, TX 77845, United States; b Vetagro S.p.A., Via Porro 2, 42124 Reggio Emilia, Italy; c Department of Animal and Food Sciences, University of Delaware, Newark, DE 19716, United States; d DIMEVET, Department of Veterinary Medical Sciences, University of Bologna, Via Tolara di Sopra 50, Ozzano Emilia, 40064 Bologna, Italy; e Vetagro Inc., 936 SW 1st Ave, Suite 878 Miami, FL 33130, United States

**Keywords:** Broiler, Botanicals, Inflammation, Salmonella, Gut

## Abstract

Inducing inflammation in response to pathogen infection is known to be an energy-intensive process. An extended state of inflammation in production animals can be detrimental to performance parameters. Here, we compare two doses of a microencapsulated thymol-based feed additive blend and two different antibiotics in the context of a *Salmonella Enterica s*ubsp. *Enterica* serovar Enteritidis (SE) challenge. A total of 500, day-old, straight run chicks (Ross 708) were placed in floor pens (50 chicks/pen) and assigned to 5 groups with 2 replicates each. The groups were all fed with basal diets, without supplementation (Control) or supplemented with: tylosin at 25 g/MT (Tylosin); neomycin sulfate at 100 g/MT (Neomycin); or a microencapsulated thymol-based blend of botanicals at inclusions of 500 g/MT (Blend 500) or 1000 g/MT (Blend 1000). All the pens were orally challenged at day 4 with SE at 10^5^ CFU/bird. At 7, 14, 21, and 35-days post hatch, ten animals from each pen were weighed and euthanized in order to culture enumerate SE in the ceca and determine SE prevalence in the liver. Gene based prevalence, gene expression, and kinome analysis were performed on jejunum samples collected at day 35. The higher dose of the tested botanical, Blend 1000, showed a statistically significant increase in final body weight relative to the other groups, indicating an energy benefit for this group. The Blend 1000 also showed a reduction in protein phosphorylation that corresponds to reduced inflammatory status that was unique compared to Blend 500 and the antibiotics. These birds also showed a clearance of orally inoculated SE demonstrating that reduced inflammation can benefit the broiler chicken in clearance of bacteria while maintaining growth.

## Introduction

Natural products have been proven as potential antibiotic alternatives for use in animal agriculture; these alternatives include, but are not limited to, organic acids, botanicals, short chain fatty acids, and bacterial fermentates (postbiotics) (Johnson et al., 2023; Liu et al., 2021; Salminen et al., 2021; Swaggerty et al., 2020b, 2022). One of the challenges of utilizing these natural compounds and, more specifically botanicals, is their delivery in the desired site of action. In fact, if not protected, almost no botanicals reach the intestine, where they are needed for their beneficial effects (Haselmeyer et al., 2015; Michiels et al., 2010; Oceľová et al., 2019). Lipid microencapsulation of feed additives allows the active ingredients to by-pass the gastric environment, thus being slowly released throughout the intestine of the animal (Piva et al., 2007). Here, we evaluate the effects of two concentrations of a thymol-based blend of botanicals that have been microencapsulated for protection and delivery to the intestine, and two antibiotics, tylosin and neomycin, in the context of a *Salmonella Enterica s*ubsp. *Enterica* Serovar Enteritidis (SE) challenge.

SE represents the most frequently reported serotype causing human salmonellosis in the United States (CDC, 2016). Salmonellosis is a common foodborne disease that causes acute gastroenteritis in humans and can be transmitted by raw or undercooked poultry products (Wessels et al., 2021). Since the large majority of chickens harboring SE are asymptomatic and, as a consequence, there is no clinical evidence of infection, cross-contamination of products at the processing plant is difficult to prevent (Grilli et al., 2011).

Botanicals are generally classified as either plant extracts, like essential oils and oleoresins, or nature-identical compounds, that represent the chemically synthetized single compounds contained in plant extracts, like thymol (Rossi et al., 2020). The existing great variety of botanicals reflects many different properties that this category of compounds can exert, such as antimicrobial, anti-inflammatory, and antioxidant. In the past, the role of several botanicals against *Salmonella* has been reviewed. During an *in vitro* challenge with *Salmonella* Typhimurium on Caco-2 cells, thymol and carvacrol showed a positive effect on both the host and the pathogen, contributing to the maintenance of the intestinal epithelial integrity and the downregulation of *Salmonella* virulence genes (Giovagnoni et al., 2020). The immunometabolic modulation exerted by four plant extracts was investigated during a SE infection on a chicken macrophage-like cell line, with different responses induced in the insulted cells (Giovagnoni et al., 2023b). Finally, the supplementation of a microencapsulated feed additive containing sorbic acid and nature-identical compounds reduced *Salmonella* Hadar and *Salmonella* Enteritidis colonization in experimentally infected chickens (Grilli et al., 2011).

In this study, in order to evaluate the host response at a protein activity level, a kinomics approach to measure phosphorylation events was employed. Phosphorylation, being the foremost means of post-translational protein modification, plays a key role in almost all cellular signaling events and the regulation of biological processes and provides valuable mechanistic insights (Manning et al., 2002). Due to the central importance of phosphorylation as a means of mediating responses to the environment and controlling cellular processes, measuring phosphorylation provides a valuable insight into changes in host physiology due to a stressor or treatment. Peptides, representing the functional targets of kinase enzymes, provide a way to characterize changes in kinomic activity (Jalal et al., 2009). Chicken-specific kinome arrays have been developed (Arsenault, 2012; Arsenault et al., 2013) and are available to analyze global kinase activity, providing insight into immunometabolic signaling changes that occur between treatment and control groups (Jalal et al., 2009; Li et al., 2012), making it possible to characterize the mode-of-action associated with a feed additive in context (Arsenault et al., 2017). Using a kinomic approach to explore the changes to intracellular signaling cascades associated with immune and metabolic processes in response to blends of bioactive compounds, we are able to better understand the changes conferred resulting in improved production measures and response to foodborne pathogen exposure (Johnson, et al., 2023; Swaggerty et al., 2022, 2020a).

The objectives of the current study were to further characterize the mechanism of action of this microencapsulated thymol-based blend of botanicals by defining the site-specific changes in phosphorylation status of proteins, measuring subsequent gene expression changes, and inferring the physiological consequences of those changes.

## Materials and methods

### *Experimental Design*

The experiments were conducted in accordance with guidelines set by the United States Department of Agriculture Animal Care and Use Committee. The trials were conducted at the Agricultural Research Service Facility of the United States Department of Agriculture (ARS-USDA), College Station, Texas, US.

A total of 500, day-old, straight run chicks (Ross 708) were placed in floor pens (50 chicks/pen) and assigned to 5 groups with 2 replicates each. The groups were all fed with basal diets, without supplementation (Control) or supplemented with: tylosin at 25 g/MT (Tylosin); neomycin sulfate at 100 g/MT (Neomycin); or a thymol-based blend of botanicals at inclusions of 500 g/MT (Blend 500) and 1000 g/MT (Blend 1000). The microencapsulated blend tested in the present study was a proprietary mixture of botanicals manufactured by Vetagro S.p.A. (Reggio Emilia, Italy).

All the pens were orally challenged at day 4 with a poultry isolate of SE at 1.4×10^5^ CFU/bird. The bacterial strain was obtained from the National Veterinary Services Laboratory (Ames, IA, USA), and was selected for resistance to nalidixic acid and novobiocin. Prior to the *in vivo* study, the susceptibility of the challenge strain to a panel of antibiotics was evaluated using the Sensititre™ NARMS Gram Negative Plate and the Sensititre™ Avian AVIAN1F Vet AST Plate (Thermo Scientific™). Among the tested antibiotics, tylosin and neomycin were chosen, as the challenge strain was resistant to the former and susceptible to the latter (data not shown). Although birds were housed in two pens per treatment group, statistical analyses were conducted using individual animals (n = 10 per pen, 20 per treatment) as observational units. Each bird was independently sampled and processed for microbiological, molecular, and kinome analyses. Environmental conditions, feed, and handling procedures were standardized across pens to minimize potential pen effects. This approach was adopted to capture biological variability among individual birds, which was central to the study objectives.

At 7, 14, 21, and 35 days of life, ten animals from each pen were weighed and euthanized in order to enumerate SE in ceca and determine its prevalence in the liver. The ceca and the liver from each chicken were removed aseptically. The cecal contents (250 mg) were diluted and spread onto XLT4 agar base plates with XLT4 supplement (Difco) and nalidixic acid and novobiocin (XLT-NN), to enumerate SE. Liver was placed in Rappaport-Vassiliadis R10 Broth (Difco) to enrich for SE and spread on XLT4 agar base plates with XLT-NN to determine SE presence. Data derived from measurement of body weight and SE enumeration in ceca were analyzed using Graphpad Prism v. 10.4.1 (GraphPad Software Inc., San Diego, CA, USA) by performing an ordinary two-way ANOVA, followed by a Tukey's multiple comparisons test to compare every treatment with each other. Differences were considered significant at p < 0.05.

At day 35, SE results between replicates of the neomycin groups were clearly different, with one replicate that showed high SE counts and one replicate that totally cleared SE. Given this unexpected divergence, and although both replicates received the same treatment under identical conditions, we performed a t-test between the two neomycin replicates on day 35 ceca counts and day 35 body weight data as an exploratory analysis. To confirm *Salmonella* infection status in all jejunum samples, samples were run on a BAX® System for *Salmonella* detection, going forward we indicated neomycin samples throughout this manuscript as Neomycin + (for chickens that were treated with neomycin but did not clear the SE infection) and Neomycin - (for chickens that were treated with neomycin and did clear the SE infection). The aim was to investigate whether the observed difference in SE clearance could be associated with variations in further analysis. This subgrouping was used solely for descriptive and exploratory purposes, and results are interpreted with appropriate caution.

### *BAX® System Salmonella Detection*

Tissue samples were removed from chickens at day 35 necropsy and immediately flash frozen in liquid nitrogen to preserve sample integrity. Six jejunum samples, matching samples used for kinome peptide array protocol, were selected from each treatment group and a modified BAX System (Hygiena LLC, Camarillo, CA, USA) protocol was carried out using BAX System’s Real-Time PCR Assay kit for *Salmonella* detection (KIT2006) (Hygiena LLC, Camarillo, CA, USA). All reagents were from the BAX System *Salmonella* Kit unless otherwise specified.

Jejunum samples of between 250 and 270 mg were placed in 2.0 mL homogenizer tubes containing 1.4 mm Ceramic beads (Fisher Scientific, Pittsburg, PA, USA) with 1 mL BAX media containing 0.5 mL/L of BAX Quant Solution and homogenized using an Omni BeadRuptor24 (Omni International, Kennesaw, GA) on setting 6 for two cycles of 20 seconds each with 5 second interval between cycles. One milliliter of the resultant slurry was transferred to a tube containing 14 mL of BAX media, subsequently, 10 mL of the mixture was added to an additional 10 mL of BAX media containing 0.5 mL/L of BAX Quant Solution. This sample preparation was incubated for 22 hours at 42°C. BAX System protocol was followed per manufacturer’s instructions for positive/negative result. A *Salmonella* positive control and a *Salmonella* negative control were included in the run as process controls to validate run integrity.

### *Kinome Peptide Arrays*

***Procedure.*** Tissue samples were removed from chickens and immediately flash frozen in liquid nitrogen to preserve kinase enzymatic activity. Samples were shipped to University of Delaware on dry ice and stored at -80°C until experimental protocol was conducted as previously described and summarized below (Arsenault, et al., 2017).

A ∼40 mg section of jejunal tissue was collected and placed in 2.0 mL homogenizer tubes containing 1.4 mm Ceramic beads (Fisher Scientific, Pittsburg, PA, USA) and 100 uL of lysis buffer (20 mM Tris-HCl, pH 7.5, 150 mM NaCl, 1 mM EDTA, 1 mM EGTA, 1% Triton, 2.5 mM sodium pyrophosphate, 1 mM Na_3_VO_4_, 1 mM NaF,1 ug/mL leupeptin, 1 g/mL aprotinin, and 1 mM phenylmethylsulfonyl fluoride [PMSF]) (all products from Sigma-Aldrich, St. Louis, MO, unless indicated). Samples were homogenized in a BeadRuptor 24 (Omni International) for two 10-sec. cycles at machine speed 6.

Homogenized tissue was spun in a refrigerated microcentrifuge at 14,000xg for 10 min. at 4°C. A 70 uL aliquot of the resultant supernatant was mixed with 10 uL of activation mix (50% glycerol, 500 uM ATP [New England BioLabs, Ipswich, MA], 60 mM MgCl2, 0.05% [vol/vol] Brij 35, 0.25 mg/mL bovine serum albumin [BSA]) in a new microcentrifuge tube. Peptide array production was done on a contract basis with JPT Peptide Technologies (Berlin, Germany). Seven hundred seventy-one unique kinase substrate target peptide sequences were printed in replicate 9 times.

A 25 × 60 mm, 85 uL glass lifter slip was applied to the microarray to sandwich and disperse the applied lysate. Eighty μL of the resulting sample mixtures were applied to the peptide microarray, ensuring that no bubbles were present in the pipette tip or array slide. Slides were incubated for 2 hrs. in a humidity chamber consisting of a sealed container containing a small amount of water (not in contact with the arrays) placed within an incubator at 37°C at 5% CO_2_. Arrays were removed from the incubator and humidity chamber and placed in a 50 mL centrifuge tube containing phosphate-buffered saline (PBS)–1% Triton X-100. The arrays were submerged repeatedly until the lifter slip lifted off the array. Arrays were then submerged in 2 M NaCl-1% Triton X-100 and agitated for a minimum of 10 seconds. This process was then repeated with fresh 2 M NaCl-1% Triton X-100. Arrays were submerged in ddH_2_0 and agitated for a minimum of 10 seconds. Array slides were removed from the ddH20 and submerged in phospho-specific fluorescent ProQ Diamond Phosphoprotein Stain (Life Technologies, Carlsbad, CA) in a dish and placed on a shaker table at 50 rpm for one hr. The dish was covered to protect the fluorescent stain from light. Arrays were then placed in a new dish and submerged in destaining solution containing 20% acetonitrile (EMD Millipore Chemicals, Billerica, MA) and 50 mM sodium acetate at pH 4.0 for 10 min with agitation at 50 rpm. The dish was covered to protect the stain from light. This process was repeated 2 times. A final wash was done with distilled deionized H_2_O. Arrays were placed in 50 mL centrifuge tubes with a crumpled kimwipe in the bottom. The tubes containing the arrays were then centrifuged at 300 x g for 2 min to remove any moisture from the array. Arrays were scanned using a Tecan PowerScanner microarray scanner (Tecan Systems, San Jose, CA) at 532 to 560 nm with a 580-nm filter to detect dye fluorescence.

***Kinome peptide array data and statistical analysis.*** Images were generated, and the spot intensity signal was collected as the mean of pixel intensity using local feature background intensity calculation with the default scanner saturation level. Images were gridded using GenePix Pro software, and the spot intensity signal collected as the mean of pixel intensity using local feature background intensity calculation with the default scanner saturation level. The resultant data was then analyzed by the PIIKA2 peptide array analysis software (http://saphire.usask.ca/saphire/piika/index.html; Trost et al., 2013). Briefly, the resulting data points were normalized using variance stabilization normalization to eliminate unequal variance between peptide spots and across arrays. Using the normalized data set, comparisons between the treatment groups and control were performed, calculating fold-change and a significance p-value. The p-value was calculated by conducting a one-sided paired t-test between treatment and control values for a given peptide. The resultant fold-change and significance values were then used to generate higher order analysis (heatmaps, hierarchical clustering, principal component analysis, pathway analysis, etc.). The kinome peptide array analysis was performed in triplicate for each group per tissue. From each experimental group samples of N = 6 kinome were collected for analysis. As described by (Perry et al., 2020), post PIIKA 2.5 analysis was performed using the following online databases and tools; STRING database (Szklarczyk et al., 2019), Reactome pathway database (Milacic et al., 2024), PhosphoSitePlus (Hornbeck et al., 2015), and Uniprot (UniProt Consortium, 2021). As the chicken-specific kinome peptide array was designed by finding orthologous peptides to known human peptides with known function within the chicken proteome and confirming the nature of those peptides within the chicken proteome, we are able to use human pathway mapping tools within this dataset (Arsenault, et al., 2014). Thus, the human Reactome Pathway IDs are presented in the pathway tables throughout the manuscript. False discovery rate (FDR) correction is applied to the STRING database pathway outputs. False discovery rate is a multiple testing correction applied for large datasets (Benjamini and Hochberg, 1995). The FDR is the rate the proteins in the indicated as significant are actually null. An FDR of 5% means that, among all proteins called significant, 5% of these are truly null. Similar to how an alpha threshold is set for p-value cut off, a similar value is set for the q-value of FDR. The q-value is the expected proportion of false positives among all proteins listed.

### *Isolation of total RNA for quantitative Real-Time RT-PCR*

Tissue samples were homogenized with a Benchmark BeadBlaster 24 homogenizer (Benchmark Scientific, Edison, NJ). Briefly, a piece jejunum (∼25 mg) was collected and placed in 2.0 mL homogenizer tubes containing 1.4 mm ceramic beads (Fisher Scientific, Pittsburg, PA, USA), lysis buffer (Maxwell RSC simplyRNA Tissue Kit; Promega, Madison, WI) was added, and the tissue was homogenized for 2 min at maximum speed. Total RNA was isolated from the homogenized tissue according to the manufacturer's instructions (Maxwell RSC simplyRNA Tissue Kit [Promega, Madison, WI]), eluted with 50 μL RNase-free water, and stored at −80°C.

### *Quantitative Real-Time RT-PCR*

The qRT-PCR was performed using the TaqMan one-step RT-PCR master mix reagents (Applied Biosystems, Branchburg, NJ). Amplification and detection of specific products were performed using the Applied Biosystems 7,500 Fast Real-Time PCR System with the following cycle profile: one cycle of 48°C for 30 min and 95°C for 20 s and 40 cycles of 95°C for 3 s and 60°C for 30 s. Quantification was based on the increased fluorescence detected by the 7,500 Fast Sequence Detection System due to hydrolysis of the target-specific probes by the 5′ nuclease activity of the rTth DNA polymerase during PCR amplification. Sample standardization was done using TATA-box binding protein (TBP) RNA. mRNA expression was normalized using TBP as housekeeping gene and results were calculated using the ddCt method (Livak and Schmittgen, 2001). Significance was calculated using JMP software version 15 (JMP Statistical Discovery Cary, NC) by ANOVA and Tukey’s post hoc test.

## Results

### Body Weights and Salmonella Detection

**Fig. 1:**
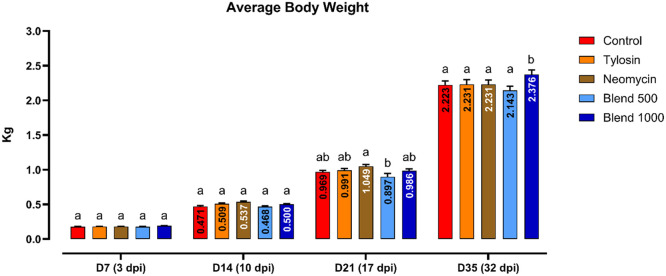
**Chicken Body Weights.** Ten birds from each pen and group were weighed at select time points through the course of the experiment. Significance was calculated by ordinary two-way ANOVA, followed by a Tukey's multiple comparisons test and differences considered significant at p<0.05. D = day, dpi = days post-infection.

The average body weight of the chickens throughout the duration of the study is reported in Fig. 1. Since no statistical difference between body weights of Neomycin + and Neomycin – at day 35 was observed (data not shown), data from the two replicates were combined. Body weights remained stable until day 21, when a statistical difference was observed between Neomycin and Blend 500 (p = 0.023). At day 35, the final body weight of Blend 1000 was significantly higher than all the other groups (+153 g compared to Control).

**Fig. 2:**
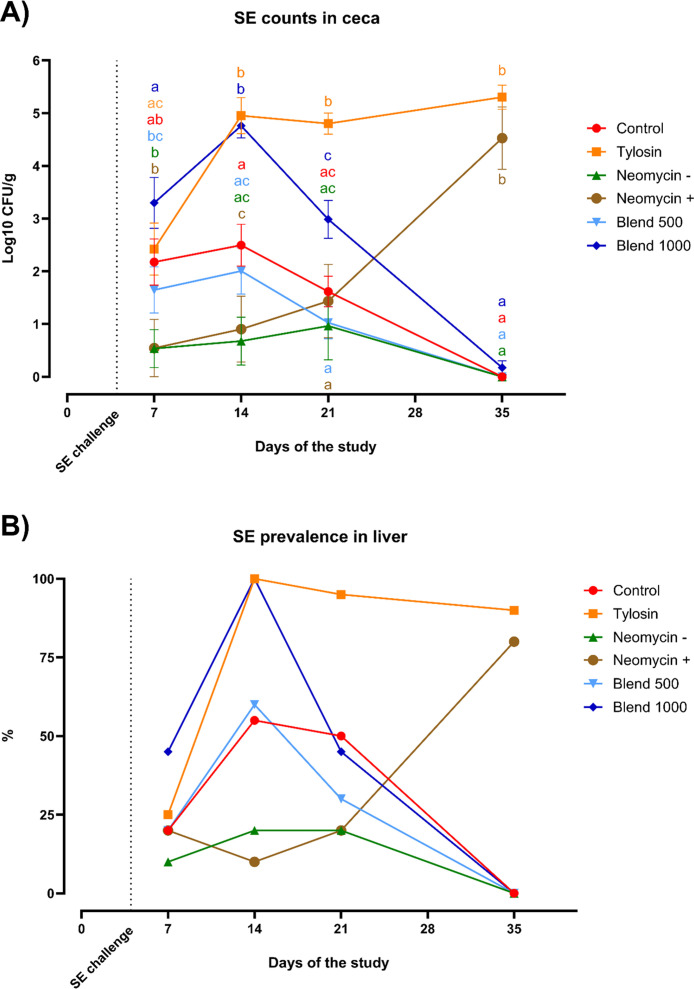
**Salmonella Counts in Ceca and Liver Prevalence.** Ten birds from each pen and group were sacrificed at select time points through the course of the experiment to count SE in ceca (A) and determine its prevalence in the liver (B). Significance was calculated by ordinary two-way ANOVA, followed by a Tukey's multiple comparisons test and differences considered significant at p<0.05.

A statistically significant difference was observed in ceca SE counts between Neomycin + and Neomycin – at day 35 (data not shown), hence the two groups were displayed separately in Fig. 2. The same trend of SE counts in ceca (Fig. 2A) was observed for SE prevalence in liver (Fig. 2B). The data for SE counts in ceca for Control, Blend 500, and Blend 1000 showed a peak at d14, followed by a progressive decrease until the bacteria were totally cleared at day 35 (Fig. 2A). Tylosin had high counts throughout the experiment, concluding with 5.3 Log10 CFU/g. The trend of Neomycin - and Neomycin + was comparable until day 21, whereas at day 35 the former totally cleared the pathogen and the latter showed presence of SE in liver and strong contaminations of ceca (4.53 Log10 CFU/g), comparable to Tylosin. The same pattern was observed in the percentage of SE prevalence in liver: at day 35 the prevalence in Control, Blend 500, Blend 1000, and Neomycin - was null, whereas in Tylosin and Neomycin + was found to be 90% and 80% respectively (Fig. 2B).

**Fig. 3:**
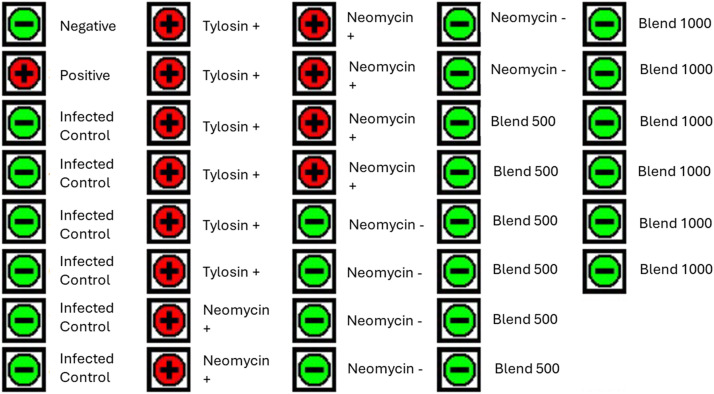
**BAX® System Salmonella detection in the jejunum.** SE prevalence in the jejunum tissue from each group utilized for kinome analysis was assayed for using the BAX® PCR-based detection system. SE presence is indicated by a red “+” and absence indicated by a green “-“.

As mentioned above, the results of SE counts in ceca and prevalence in liver (Fig. 2) indicated a different trend between the two replicates treated with neomycin. For this reason, the jejunum gut tissue was assayed using the BAX® System PCR based pathogen detection in order to confirm SE presence or absence in the jejunum tissues that underwent further analysis. The BAX® System results agreed with the culture results, Tylosin and Neomycin + tested positive for SE at day 35 while Control, Neomycin -, Blend 500 and Blend 1000 tested negative (Fig. 3). These results were in agreement with the bacterial culture results from the liver samples taken in the case of positive SE detection.

### Kinome Analysis

**Fig. 4:**
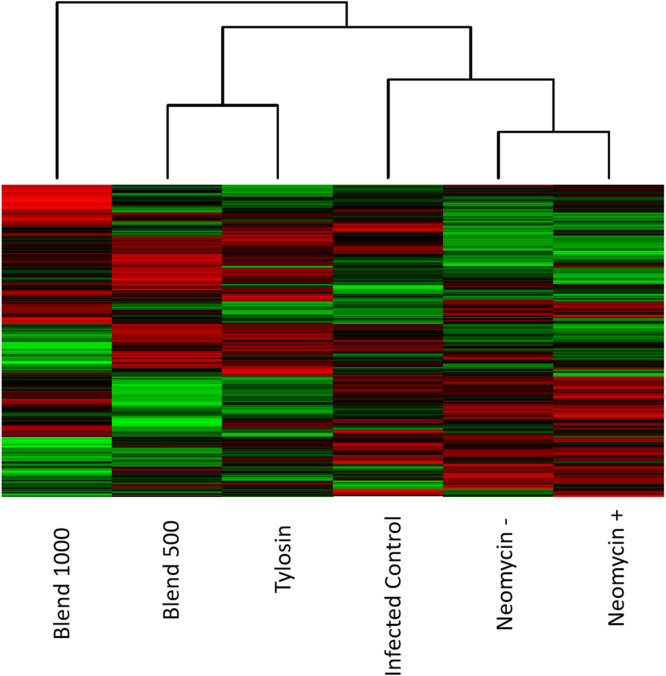
**Heat map of the phosphorylation status of the peptides represented on the array.** Red indicates increased phosphorylation and green indicates decreased phosphorylation relative to the average of the total signal. Here, the heat map shows tighter clustering between the Neomycin groups and the Blend 500 and Tylosin. Infected control is outside of these two groups and Blend 1000 is an outlier.

The kinome peptide array analysis was performed on jejunum samples collected at day 35, broken down into the 6 groups listed above, including splitting the Neomycin group into those birds in the groups that tested negative for SE at day 35 (Neomycin -) and those that tested positive (Neomycin +). Clustering of the kinome data between groups showed that the two closest groups were the two Neomycin groups, followed by the Blend 500 and Tylosin groups (Fig. 4). The infected control group clustered between the two previously mentioned clusters. Blend 1000 was an outlier compared to all the other groups and had a clearly distinct kinome profile.

**Fig. 5:**
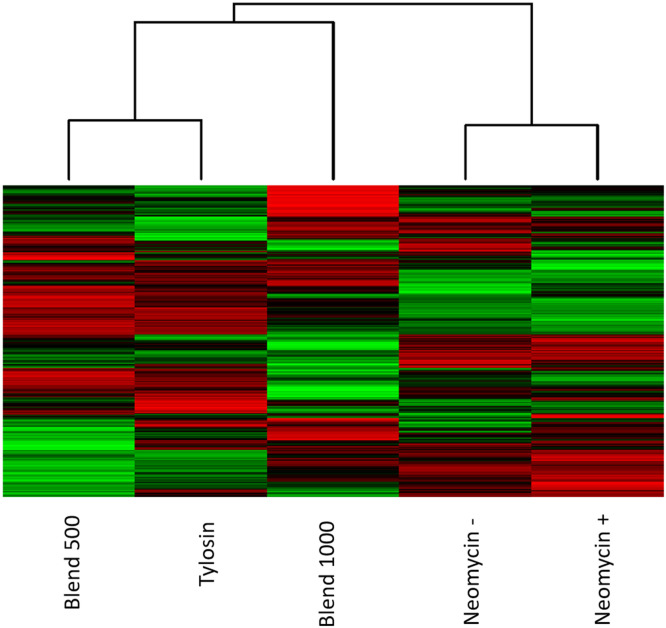
**Heat map of the phosphorylation status of the peptides represented on the array compared to infected control.** Phosphorylation signal from the treatment groups was compared to infected control to create a fold change. Red indicates increased phosphorylation and green indicates decreased phosphorylation relative to infected Control. The heat map shows tighter clustering between the Neomycin groups and the Blend 500 and Tylosin. Blend 1000 is an outlier.

In order to represent the data as a relative increase or decrease in phosphorylation compared to responses related to SE infection, all treatment group data was compared to infected Control to generate a phosphorylation fold change and significance value for each peptide. The clustering of this phosphorylation relative to Control is shown in Fig. 5. The trend is quite similar to Fig. 4, where just the normalized signal is represented, in that the Neomycin groups cluster and the Blend 500 and Tylosin cluster. The Blend 1000 remains distinct from these two groupings.

The heatmap results indicate that the tissue exposed to Neomycin in the context of SE exposure show similar phosphorylation patterns, irrespective of if SE is detectably present. Blend 500 appears to act most similarly to Tylosin than the other groups, while Blend 1000 shows signaling distinct from the antibiotics and the lower dose of the Blend. There are dose dependent effects that are only observed in the tissue signaling when the higher dose was provided.

**Fig. 6:**
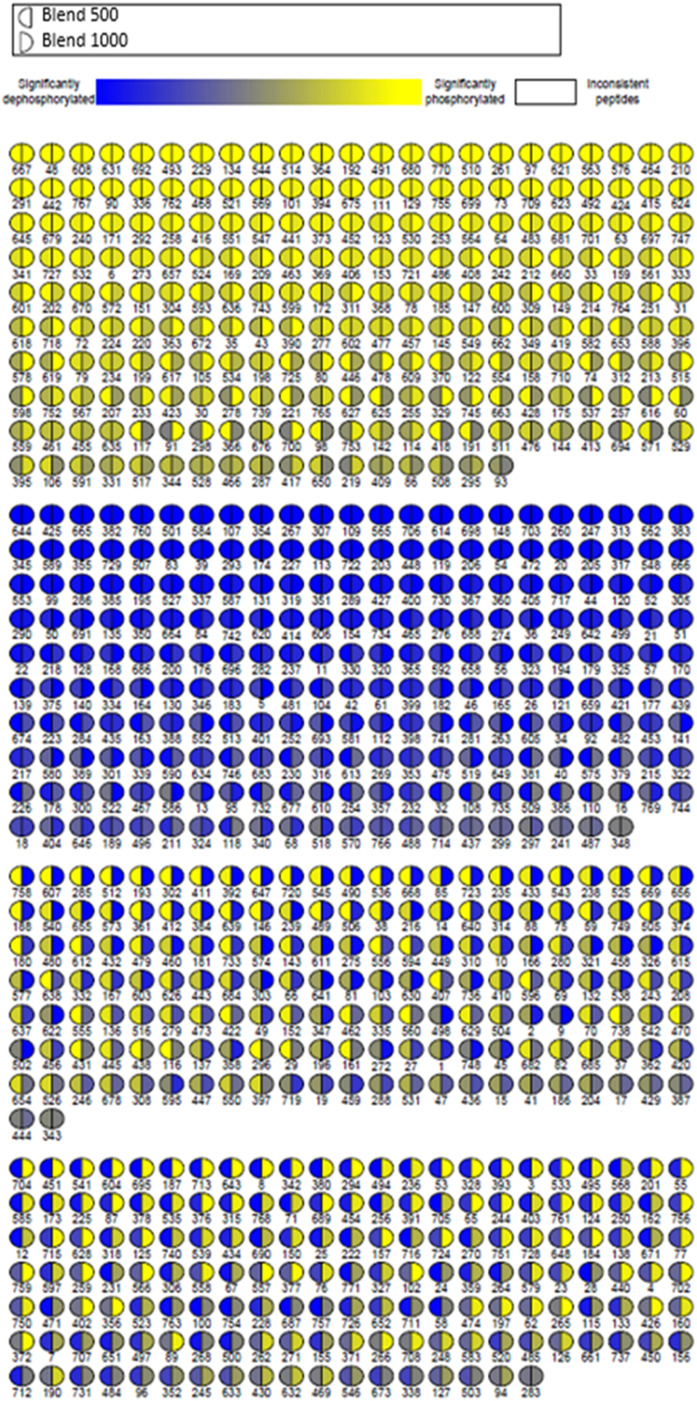
**Phosphorylation visualization, Blend 500 and Blend 1000.** Relative peptide phosphorylation significance vs SE infected positive control for Blend 500 (left side) and Blend 1000 (right side) groups are represented as circles. The two groups generate quite different phosphorylation profiles.

In order to better characterize the dose dependent differences between Blend 500 and Blend 1000 we generated a visualization map showing the phosphorylation state of each peptide for each group (Fig. 6). Each circle represents a peptide on the kinome peptide array, the left half of each circle is a color representation of the phosphorylation status of that peptide in the Blend 500 data, the right half of the circle represents the Blend 1000. As was expected from the heatmap figures, the two doses produced quite distinct phosphorylation profiles. There is a relatively large number of peptides in the bottom two blocks, which show opposite direction of phosphorylation between the two groups. This indicates that there is a significant dose dependent effect that generates novel physiological responses depending on the dose.

The statistically significantly differentially phosphorylated peptides from each treatment group relative to Control were input into the STRING protein-protein interaction database. STRING generates networks and lists of overrepresented pathways from these datasets. Table 2, Table 3, Table 4, Table 5, Table 6 below show the Reactome (Milacic et al., 2024) pathways overrepresented in each respective dataset. Innate immune related pathways are highlighted in each table.

The Table 2, Table 3, Table 4, Table 5, Table 6 above show several innate immune related pathways being significantly over-represented in the kinome data for each group. It is important to remember that all of the phosphorylation fold-changes for each treatment group were relative to Control. Thus, each of the treatment groups is having a distinct effect on innate immune signaling from that induced by SE alone. In order to determine the directionality of these changes and the ultimate effect on pathway activation and physiological response within these groups, the individual proteins and peptides phosphorylation state had to be considered.

We took the proteins found in each of the highlighted pathways in Tables 2 through 6 and identified the protein members and peptides in the peptide array data. We eliminated proteins solely related to metabolism in order to focus specifically on the immune and inflammatory response. Using the PhosphoSitePlus database (Hornbeck et al., 2015) to functionally annotate the observed kinome data we could determine the functional consequences of differential phosphorylation on each protein relative to Control. Table 7 shows these results and are color coded based on putative increased or decreased activity or transcription activation. Red indicates increased protein activity, green decreased protein activity, orange increased protein activity leading to transcription activation, and blue decreased protein activity leading to lower transcription activation.

### Cytokine Gene Expression

Jejunum tissue from birds of each experimental group were assayed for select cytokine expression levels (Table 1). Statistically significant differences in expression compared to Control was observed for IL-1β, TGF-β4 and IL-22. The Neomycin - group showed a 2.47-fold increase in expression of IL-1β. The Blend 500 and Blend 1000 groups showed a 16.01 and 14.61-fold increase in TGF-β4, respectively. The Neomycin - group and the Blend 1000 groups showed a –3.03 and –3.34-fold change reduction in IL-22 expression, respectively.

**Table 1:** PCR Primer Probe Sets.

RNA Target	Probe/Primer Sequence		Accession	Source
TBP	Probe	5′-(FAM)-CCCAGCTCTTCCACTCACAGACTC-(TAMRA)-3′	NM_001396191.1	Designed using IDT PrimerQuest™ Tool
	Forward	5′-CAGGGAACATCTGGTCAAACT-3′		
	Reverse	5′-GCAGGAGTTATAGGAGTCATTGG-3′		
IL-1β	Probe	5′-(FAM)-CCACACTGCAGCTGGAGGAAGCC-(TAMRA)-3′	NM_204524.2	(Kaiser et al., 2000)
	Forward	5′-GCTCTACATGTCGTGTGTGATGAG-3′		
	Reverse	5′-TGTCGATGTCCCGCATGA-3′		
IL-22	Probe	5′-(FAM)-CAGGCTTGATGGGCATTGGAAACC-(TAMRA)-3′	NM_001199614.1	Designed using IDT PrimerQuest™ Tool
	Forward	5′-CTGTTGTTGCTGTTTCCC-3′		
	Reverse	5′-GCGATTCCTGATGTAGGG-3′		
TGF-β4	Probe	5′-(FAM)-ACCCAAAGGTTATATGGCCAACTTCTGCAT-(TAMRA)-3′	NM_001318456.1	(Rothwell et al., 2004)
	Forward	5′-AGGATCTGCAGTGGAAGTGGAT-3′		
	Reverse	5′-CCCCGGGTTGTGTTGGT-3′		

## Discussion

Inducing inflammation in response to pathogen infection is known to be an energy intensive process (Broom and Kogut, 2018). Extended states of inflammation in production animals can result in decreased performance. Here, we observed that the higher dose of the tested thymol-based blend of botanicals, Blend 1000, showed a statistically significant increase in body weight relative to the other groups (Fig. 1), indicating an energy benefit imparted by the higher dose of the blend. The effect of the Blend 1000 also showed a reduction in protein phosphorylation induced inflammatory status, also supported by an increase of the expression of TGF-β4 and a reduction of IL-22 gene expression. These birds showed a clearance of orally inoculated SE demonstrating that reduced inflammation can benefit the broiler by maintaining growth during the clearance of bacteria. With B1000 we have a beneficial effect on final BW, together with a clearance of the foodborne pathogen probably due to its mechanism of action. Importantly, this combination of effects, including improved final body weight, reduced inflammation, and SE clearance, was obtained only in this feed additive group, and not in the animals treated with antibiotics or in the lower-dose blend group. SE is known to take advantage of the host's inflammatory response to invade across the epithelial barrier of the gut into organs such as the liver (Shaji et al., 2023). A reduction in inflammation may reduce the ability of SE to invade and persist. Alternatively, a reduction in inflammation could reduce a proven effective method of SE reduction and control. However, simply any type of anti-inflammatory response is not necessarily positive, the tylosin-treated group showed persistent SE colonization throughout the experimental time course. The appropriate effect on inflammation at the appropriate time is likely key to reducing the SE burden in chickens. Tylosin and Neomycin+ groups showed similar gene expression results and failed to clear SE at the end of the study. These findings suggest that the type of immune modulation induced by Tylosin, and partially by Neomycin, may not have favored effective pathogen clearance.

From the phosphorylation data presented here, the animals showing a persistent SE presence displayed a greater inflammatory response than any of the treatment groups, antibiotic or blend at either dose. Put another way, all of the treatment groups displayed reduced inflammation relative to the infected control group. Although SE clearance was also observed in the control and other groups by the end of the trial, the distinctive value of Blend 1000 lies in the concurrent presence of SE clearance, improved growth performance, and reduced inflammation, none of which were simultaneously achieved in any other group. The clearance of SE observed in the control group by day 35, may reflect the natural resolution of infection under low-stress experimental conditions. In commercial settings, stressors before slaughter, such as feed withdrawal or transport, are known to exacerbate microbial shedding and prolong colonization. However, in a controlled environment, birds are not exposed to such stressors, which may have facilitated spontaneous clearance.

There is a significant and obvious dose dependent response at the protein phosphorylation level between the Blend 500 and the Blend 1000 doses. There are unique effects that occur within the gut tissue of chickens that are only observed in the higher doses. Indications from both the growth of the birds in the group and from the unique protein phosphorylation signaling patterns observed here indicate that this may be beneficial in the context of growth during SE exposure. Within the immune and inflammatory protein list we see that Blend 1000 is distinct from the other groups in the activation status of certain proteins (Table 7), this distinction was observed in the heat maps as well (Fig. 4, Fig. 5). When we compare the directionality of phosphorylation of the individual peptides, we see that Blend 500 and Blend 1000 are quite distinct in the directionality of phosphorylation of the individual peptides, with nearly half of the peptides on the array showing differences between the two groups (Fig. 6).

When we consider the overrepresented pathways generated from the statistically significantly differentially phosphorylated proteins in each treatment group, we observe that there is significant overlap in the immune pathways (Table 2, Table 3, Table 4, Table 5, Table 6). All of the groups show significant changes in pathways such as immune system, cytokine signaling in immune system, and Toll-like receptor cascades. The Blend 1000 group shows additional immune signaling pathways that are unique including, MyD88:MAL(TIRAP) cascade initiated on plasma membrane, Toll-Like Receptor 9 (TLR9) Cascade, TRAF6 mediated induction of NF-κB and MAP kinases upon TLR7/8 or 9 activation, MyD88 cascade initiated on plasma membrane, MyD88 cascade initiated on plasma membrane, and TRIF(TICAM1)-mediated TLR-4 signaling (Table 3). These additional immune signaling pathways in the top hits indicate a more pronounced effect on the innate immune system due to the Blend 1000 in the chicken jejunum.

**Table 2:** Blend 500 Overrepresented Pathways.

Reactome Pathway ID	Pathway description	Observed protein count	Strength	False discovery rate (FDR)
**HSA-168256**	**Immune System**	**93**	**0.64**	**1.11E-32**
HSA-9006934	Signaling by Receptor Tyrosine Kinases	53	0.98	7.23E-32
**HSA-1280215**	**Cytokine Signaling in Immune system**	**59**	**0.9**	**9.44E-32**
HSA-162582	Signal Transduction	104	0.54	4.74E-30
HSA-1643685	Disease	78	0.66	2.21E-28
HSA-5663202	Diseases of signal transduction by growth factor receptors and second messengers	44	1.01	2.47E-27
**HSA-449147**	**Signaling by Interleukins**	**43**	**0.95**	**1.78E-24**
**HSA-168249**	**Innate Immune System**	**58**	**0.71**	**1.83E-22**
HSA-5683057	MAPK family signaling cascades	35	1	3.93E-21
HSA-187037	Signaling by NTRK1 (TRKA)	24	1.28	7.76E-20
HSA-9006925	Intracellular signaling by second messengers	33	0.99	8.96E-20
HSA-166520	Signaling by NTRKs	25	1.23	9.46E-20
**HSA-168898**	**Toll-Like Receptor Cascades**	**25**	**1.18**	**1.33E-18**
HSA-5684996	MAPK1/MAPK3 signaling	30	0.99	8.67E-18
HSA-5673001	RAF/MAP kinase cascade	29	0.98	4.79E-17
HSA-4420097	VEGFA-VEGFR2 Pathway	20	1.27	1.98E-16
HSA-2219528	PI3K/AKT Signaling in Cancer	19	1.22	1.11E-14
**HSA-166016**	**Toll-Like Receptor 4 (TLR4) Cascade**	**20**	**1.16**	**1.52E-14**
HSA-1257604	PIP3 activates AKT signaling	25	0.94	1.30E-13
HSA-187687	Signalling to ERKs	13	1.54	2.16E-13
**HSA-168164**	**Toll-Like Receptor 3 (TLR3) Cascade**	**17**	**1.23**	**2.91E-13**

Innate immune related pathways are highlighted.

**Table 3:** Blend 1000 Overrepresented Pathways.

Reactome Pathway ID	Pathway description	Observed protein count	Strength	False discovery rate (FDR)
**HSA-168256**	**Immune System**	**91**	**0.61**	**3.29E-29**
HSA-9006934	Signaling by Receptor Tyrosine Kinases	51	0.95	9.46E-29
**HSA-168249**	**Innate Immune System**	**67**	**0.75**	**2.30E-28**
HSA-162582	Signal Transduction	102	0.51	1.54E-26
HSA-5663202	Diseases of signal transduction by growth factor receptors and second messengers	44	0.99	2.21E-26
HSA-1643685	Disease	76	0.63	2.14E-25
**HSA-449147**	**Signaling by Interleukins**	**43**	**0.93**	**1.23E-23**
**HSA-1280215**	**Cytokine Signaling in Immune system**	**51**	**0.81**	**1.41E-23**
HSA-5683057	MAPK family signaling cascades	34	0.97	1.88E-19
HSA-2219528	PI3K/AKT Signaling in Cancer	23	1.28	6.44E-19
HSA-9006925	Intracellular signaling by second messengers	32	0.96	3.80E-18
**HSA-168898**	**Toll-Like Receptor Cascades**	**25**	**1.16**	**4.41E-18**
**HSA-166058**	**MyD88:MAL(TIRAP) cascade initiated on plasma membrane**	**20**	**1.27**	**3.47E-16**
**HSA-168138**	**Toll-Like Receptor 9 (TLR9) Cascade**	**20**	**1.27**	**3.47E-16**
HSA-187037	Signaling by NTRK1 (TRKA)	21	1.2	5.39E-16
**HSA-975138**	**TRAF6 mediated induction of NFkB and MAP kinases upon TLR7/8 or 9 activation**	**19**	**1.27**	**1.64E-15**
HSA-5684996	MAPK1/MAPK3 signaling	28	0.94	1.93E-15
**HSA-166016**	**Toll-Like Receptor 4 (TLR4) Cascade**	**21**	**1.16**	**2.20E-15**
HSA-1257604	PIP3 activates AKT signaling	27	0.95	3.91E-15
HSA-4420097	VEGFA-VEGFR2 Pathway	19	1.23	5.07E-15
HSA-5673001	RAF/MAP kinase cascade	27	0.93	7.96E-15
**HSA-975871**	**MyD88 cascade initiated on plasma membrane**	**18**	**1.27**	**7.96E-15**
**HSA-168164**	**Toll-Like Receptor 3 (TLR3) Cascade**	**18**	**1.23**	**2.73E-14**
**HSA-937061**	**TRIF(TICAM1)-mediated TLR4 signaling**	**18**	**1.21**	**5.11E-14**

Innate immune related pathways are highlighted.

**Table 4:** Tylosin Overrepresented Pathways.

Reactome Pathway ID	Pathway description	Observed protein count	Strength	False discovery rate (FDR)
HSA-9006934	Signaling by Receptor Tyrosine Kinases	47	1.04	7.13E-31
HSA-162582	Signal Transduction	86	0.57	3.40E-27
HSA-1643685	Disease	63	0.68	7.41E-24
**HSA-168256**	**Immune System**	**69**	**0.62**	**2.75E-23**
**HSA-1280215**	**Cytokine Signaling in Immune system**	**44**	**0.88**	**4.53E-23**
HSA-5663202	Diseases of signal transduction by growth factor receptors and second messengers	35	1.02	3.82E-22
**HSA-449147**	**Signaling by Interleukins**	**34**	**0.96**	**1.24E-19**
HSA-9006925	Intracellular signaling by second messengers	27	1.02	1.15E-16
**HSA-168249**	**Innate Immune System**	**44**	**0.7**	**1.16E-16**
HSA-5683057	MAPK family signaling cascades	27	1	2.89E-16
HSA-512988	Interleukin-3, Interleukin-5 and GM-CSF signaling	15	1.57	6.69E-16
HSA-1280218	Adaptive Immune System	36	0.76	6.97E-15
HSA-166520	Signaling by NTRKs	19	1.22	6.97E-15
HSA-187037	Signaling by NTRK1 (TRKA)	18	1.27	9.35E-15
HSA-76002	Platelet activation, signaling and aggregation	23	1.02	3.42E-14
HSA-5684996	MAPK1/MAPK3 signaling	23	0.99	1.42E-13
HSA-4420097	VEGFA-VEGFR2 Pathway	16	1.29	2.12E-13
HSA-912631	Regulation of signaling by CBL	11	1.77	2.13E-13
HSA-1227986	Signaling by ERBB2	13	1.49	5.46E-13
HSA-5673001	RAF/MAP kinase cascade	22	0.98	7.38E-13
HSA-9607240	FLT3 Signaling	12	1.57	7.64E-13
HSA-109582	Hemostasis	29	0.75	8.21E-12
HSA-2219528	PI3K/AKT Signaling in Cancer	15	1.23	8.21E-12
HSA-70171	Glycolysis	13	1.33	2.50E-11
HSA-2730905	Role of LAT2/NTAL/LAB on calcium mobilization	9	1.8	3.92E-11
HSA-1433557	Signaling by SCF-KIT	11	1.48	6.03E-11
**HSA-168898**	**Toll-Like Receptor Cascades**	**16**	**1.09**	**7.18E-11**

Innate immune related pathways are highlighted.

**Table 5:** Neomycin - Overrepresented Pathways.

Reactome Pathway ID	Pathway description	Observed protein count	Strength	False discovery rate (FDR)
**HSA-168249**	**Innate Immune System**	**59**	**0.76**	**7.76E-25**
**HSA-168256**	**Immune System**	**78**	**0.6**	**8.60E-25**
**HSA-1280215**	**Cytokine Signaling in Immune system**	**44**	**0.81**	**8.10E-20**
**HSA-449147**	**Signaling by Interleukins**	**37**	**0.93**	**8.10E-20**
HSA-162582	Signal Transduction	82	0.48	1.02E-18
HSA-9006934	Signaling by Receptor Tyrosine Kinases	36	0.86	2.24E-17
HSA-5683057	MAPK family signaling cascades	29	0.96	3.03E-16
HSA-1643685	Disease	57	0.57	1.06E-15
HSA-5663202	Diseases of signal transduction by growth factor receptors and second messengers	30	0.89	4.79E-15
HSA-187037	Signaling by NTRK1 (TRKA)	19	1.22	1.45E-14
HSA-2454202	Fc epsilon receptor (FCERI) signaling	19	1.17	8.80E-14
HSA-5684996	MAPK1/MAPK3 signaling	24	0.93	6.61E-13
HSA-1430728	Metabolism	60	0.46	4.51E-12
**HSA-168898**	**Toll-Like Receptor Cascades**	**18**	**1.07**	**1.21E-11**
HSA-9006925	Intracellular signaling by second messengers	23	0.88	2.42E-11
HSA-5673001	RAF/MAP kinase cascade	22	0.91	2.59E-11
**HSA-166016**	**Toll-Like Receptor 4 (TLR4) Cascade**	**16**	**1.11**	**9.61E-11**
HSA-5621481	C-type lectin receptors (CLRs)	16	1.07	2.63E-10
**HSA-168164**	**Toll-Like Receptor 3 (TLR3) Cascade**	**14**	**1.18**	**2.69E-10**

Innate immune related pathways are highlighted.

**Table 6:** Neomycin + Overrepresented Pathways.

Reactome Pathway ID	Pathway description	Observed protein count	Strength	False discovery rate (FDR)
HSA-1643685	Disease	59	0.69	7.54E-23
HSA-5663202	Diseases of signal transduction by growth factor receptors and second messengers	35	1.06	7.54E-23
HSA-9006934	Signaling by Receptor Tyrosine Kinases	37	0.98	4.26E-22
HSA-162582	Signal Transduction	74	0.54	1.30E-21
**HSA-1280215**	**Cytokine Signaling in Immune system**	**38**	**0.86**	**4.80E-19**
**HSA-168256**	**Immune System**	**60**	**0.6**	**4.80E-19**
**HSA-449147**	**Signaling by Interleukins**	**32**	**0.97**	**6.34E-19**
HSA-5683057	MAPK family signaling cascades	28	1.06	2.26E-18
HSA-9006925	Intracellular signaling by second messengers	26	1.04	1.02E-16
**HSA-168249**	**Innate Immune System**	**41**	**0.71**	**6.04E-16**
HSA-5673001	RAF/MAP kinase cascade	24	1.05	1.30E-15
HSA-2219528	PI3K/AKT Signaling in Cancer	16	1.3	1.87E-13
HSA-9006335	Signaling by Erythropoietin	11	1.76	2.91E-13
HSA-199418	Negative regulation of the PI3K/AKT network	16	1.27	4.05E-13
HSA-187037	Signaling by NTRK1 (TRKA)	16	1.26	5.52E-13
HSA-1257604	PIP3 activates AKT signaling	21	1.01	6.79E-13
**HSA-168164**	**Toll-Like Receptor 3 (TLR3) Cascade**	**14**	**1.29**	**8.55E-12**
HSA-212436	Generic Transcription Pathway	38	0.61	8.93E-12
HSA-4420097	VEGFA-VEGFR2 Pathway	14	1.27	1.49E-11
**HSA-168898**	**Toll-Like Receptor Cascades**	**16**	**1.13**	**2.10E-11**

Innate immune related pathways are highlighted.

There is also some indication that the blends may have induced a greater adaptive immune response compared to infected control. We observe indications that glycolysis was reduced (and thus inflammation) in the treatment groups relative to Control most clearly indicated by the dephosphorylation of HIF1A at a site which would reduce transcriptional activation of glycolytic enzymes (Table 7). These findings are in line with previous research relative to the effect of the thymol-based blend on SE infected HD11 cells, where treated macrophages showed lower extracellular acidification rate, that is proportional to cellular glycolysis (Giovagnoni et al., 2023a). The proteins that show a unique phosphorylation status in Blend 1000 compared to the other groups including ATF2, BTK, FLT3, ITK, Jun, LCK, PTK2, PTK2B, PTPRC and STAT1, taken together these represent and adaptive immune response, possibility related to T-cell signaling.

**Table 7:** Immune and Inflammatory Peptides of Known Function Differentially Phosphorylated Relative to *Salmonella* Infection.

			Blend 1000		Blend 500		Neomycin -		Neomycin +		Tylosin	
Uniprot ID^1^	Protein Name	Phosphosite^2^	FC^3^	P^4^	FC	P	FC	P	FC	P	FC	P


1 UniProt protein identifier

2 The amino acid site that is modified by phosphorylation

3 Fold Change

4 P-value Red indicates increased protein activity, green decreased protein activity, orange increased protein activity leading to transcription activation, and blue decreased protein activity leading to lower transcription activation.

Of note is NF-κB, a transcription factor inducing expression of pro-inflammatory proteins, this is deactivated in the Blend 1000 as well as in two of the antibiotic groups. Other sites on NF-κB in the Blend 1000 are decreased but their function is unknown in the literature. The cytokine gene expression results provide further context to the physiology of the gut under the various experimental conditions.

**Fig. 7:**
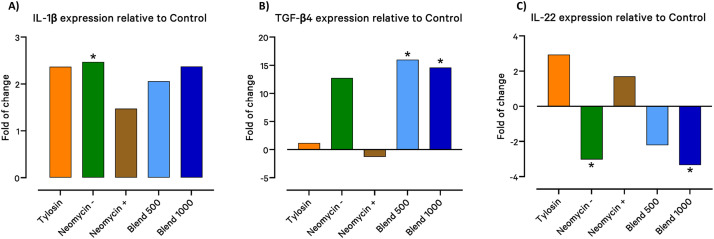
**Cytokine gene expression.** Gene expression of key cytokines was measured in birds from each group and compared to the expression of control infected birds using the ddCt method. ANOVA of Ct values followed by Tukey’s Honest Significant Difference test was conducted. Statistically significant differences were found in the expression of A) IL-1β, B) TGF-β4 and C) IL-22.

TGF-β4 cytokine plays a role in cell differentiation, survival and inflammation, it is also involved in immune tolerance (Johnston et al., 2016). In the jejunum tissue, both concentrations of the Blend product resulted in a large and statistically significant increase in TGF-β4 expression relative to control (Fig. 7B). This may indicate a more homeostatic status in the gut generated by the product. Tylosin and Neomycin + showed little to no expression relative to Control, and these tissues were still positive for SE presence at this time, perhaps indicating an active response to the infection and a lack of homeostasis. Interestingly the Control birds, which had cleared the infection but were not treated, were not generating these high levels of TGF-β4 compared to the Blend groups.

IL-22 has diverse functions throughout the host and has been shown to have both inflammatory and anti-inflammatory properties (Dudakov et al., 2015). Specifically in the gut, IL-22 has been shown to be involved in the innate immune response to enteric bacterial infection, barrier integrity and inflammation (Sonnenberg et al., 2011). Increased expression of IL-22 was observed to be induced by SE infection in chicks infected on days 1, 4, and 16 of life (Crhanova et al., 2011). Both the Neomycin - and Blend 1000 groups showed a statistically significant decrease in IL-22 expression relative to Control (Fig. 7C). Neither Neomycin - nor Blend 1000, nor Control jejuna were positive for SE at this time, yet both groups showed a reduction in expression. Blend 500 showed a numerical reduction in expression, while Tylosin and Neomycin + showed a numerical increase, continuing the trend of tissue positive for SE expressing higher levels of IL-22 while tissues negative for SE showing less expression relative to Control. Without SE present in the gut there is no need for an inflammatory or barrier enhancing response, and resolution of this response is greater with exposure to Neomycin - and Blend 1000 than without.

While all groups showed a numerical increase in gene expression of IL-1β relative to Control, only the Neomycin - group was statistically significant (Fig. 7A). It is interesting that this antibiotic treated group, which had cleared the SE infection in the gut, still displayed an elevated level of this pro-inflammatory cytokine (Dinarello, 1989).

In general, cytokine gene expression results are in line with previous findings, where the thymol-based blend showed an anti-inflammatory action on SE infected HD11 cells: when compared with an untreated control, the blend showed a tendency in increase the expression of IL-10 and significantly decreased IL-6 expression (Giovagnoni et al., 2023a).

The results presented here confirm previous studies showing that a reduction in inflammation is beneficial in controlling SE challenge. We also show that a thymol-based blend of botanicals can produce this reduction in inflammation by reducing key inflammation signaling intermediates via phosphorylation changes. This is likely the mechanism of improved growth in the Blend 1000 treated birds. Finally, we show that botanicals can generate profoundly different host responses depending on the dose applied, highlighting how critical obtaining the correct dose and targeting that dose to the correct gastrointestinal sites.

## CRediT authorship contribution statement

**Casey N. Johnson:** Data curation, Formal analysis, Investigation, Methodology, Validation, Visualization, Writing – original draft, Writing – review & editing. **Giulia Giovagnoni:** Conceptualization, Data curation, Formal analysis, Investigation, Methodology, Writing – original draft, Writing – review & editing. **Famatta Perry:** Investigation, Methodology. **Benedetta Tugnoli:** Conceptualization. **Denise Caldwell:** Investigation, Methodology. **Christina Swaggerty:** Conceptualization, Investigation, Methodology, Writing – review & editing. **Haiqi He:** Methodology. **Andrea Piva:** Conceptualization, Resources. **Ester Grilli:** Conceptualization, Resources. **J. Allen Byrd:** Investigation. **Ryan J. Arsenault:** Conceptualization, Data curation, Formal analysis, Investigation, Methodology, Writing – original draft, Writing – review & editing.

## Disclosures

A.P. is a member of the board of directors of Vetagro S.p.A. E.G. serves as an assistant professor at the University of Bologna and is a member of the board of directors of Vetagro Inc. B.T. and G.G. are employed at Vetagro S.p.A. All the other authors declare no actual or potential conflict of interest, financial or otherwise. Financial support for this research was provided by Vetagro S.p.A. (Reggio Emilia, Italy).
